# The Annual Conference and Exhibition

**Published:** 1915-07-03

**Authors:** 


### NATIONAL ASSOCIATION FOR THE FEEBLE-MINDED.

### The Annual Conference and Exhibition.

This year's Conference of the National Association for the Feeble-Minded was held on June 25 in the Council Chamber of the Guildhall of the City of London. There was a gathering of some five hundred delegates from the provincial and other authorities concerned with the carrying out of the work under the Mental Deficiency Act.

The Conference was convened at the wish of a number of the Medical Committee and others, the chairman, Sir Bryan Donkin, M.D., taking an active personal interest. It is an attempt to further the working of the Mental Deficiency Act by endeavouring to obtain a clearer common understanding amongst medical men and others as to the whole question of diagnosis, grading, and the tendering of evidence in regard to the mentally defective under the Mental Deficiency Act. It was felt that a general discussion, amongst those who are most actively participating in the working of the Mental Deficiency and other Acts, would be likely to facilitate the carrying into effect the work of the various authorities in different localities.

There was an additional interest to the proceedings this year, since the war has a special interest for this Association. The curative value of military discipline was alluded to in Dr. Potts' paper (see p. 295). On the other hand, it appears that many "mentally defectives" have been enlisted, only to be at once invalided back for prolonged and frequently very costly treatment. And the question how such low-grade cases, numbers of whom exist unrecognised in our midst, should be prevented from enlisting is obviously an important one.

The Lord Mayor, in opening the Conference, said: I am here this morning to offer a very hearty welcome to the members of the Conference. It would ill become me—a layman—to put my views forward on this subject. I do not think, however, that anyone can overrate the importance of the subject. One result of this nerve-shattering war will be found in future generations. I wish every success to your deliberations. The Lord Mayor then withdrew, after a hearty vote of thanks had been accorded him.

The President, Sir Bryan Donkin, M.D., then delivered his address, which appears on the following page.

### The Morning's Papers.

Dr. Robert Hughes, school medical officer, Stoke-on-Trent, being away at the Front, his paper on "A Scheme for the Detection and Treatment of Mentally Defective Children" was read by deputy; and was followed by Mr. W. Winch, District Inspector of Schools, who spoke not as a medical man, but as a psychologist, with a paper on "The Detection on the Large Scale of Mental Deficiency in School Children."

Dr. Boulenger, of Brussels, who spoke in French, read a paper on "The Employment of the Binet-Simon Tests in the Examination of Abnormal English and Belgian Children." The morning session was concluded with a paper by Dr. Allan Warner, medical officer, Leicester Education Committee, on "The Value of a Uniform Examination of the Feeble-Minded for Educational Purposes."

A short discussion, initiated by Dr. Shuttleworth, followed, and Sir William Chance, chairman of the Association, made the interesting announcement that Lady Stamford, Lady Llangattock, Sir George Savage, Mr. W. H. Dickinson, himself, and others had arranged to provide a challenge cup or cups in the future "for the exhibits," as an appreciation and incentive to the various homes and institutions that contributed to their annual exhibition.

### The Afternoon Session.

At the afternoon session Dr. R. Langdon Down read a paper on "Practical Application of the Binet Tests."

Dr. Charles Goring, deputy medical officer, Brixton Prison, read a paper on "The Characteristics and Identification of the Feeble-Minded Criminal." Dr. Goring, with his large experience as medical officer, at one time and another, to many of our prisons, remarked on the difficulty of defining a "defective," although we all know one when we see one. There are no specific signs or symptoms by which mental deficiency can be diagnosed. Coming from the individual to people collectively, "mental defectives" are highly differentiated from other criminals. The only test is "conduct"; he thinks like a "defective," he acts like a "defective."

One prisoner, who absolutely refused to do any kind of work whatever, even to dress himself, in reply to a question answered, "No, I am not going to do any work. Do you think I came into prison to work? I can get plenty of that outside!"

A paper by Dr. H. W. Sinclair, school medical officer to the Essex Education Committee, on "Classification of the Mentally Defective from an Administrative Point of View," concluded the afternoon session, and the Conference closed with the usual votes of thanks.

A copy of the Conference Report, containing the various papers, can be obtained from the National Association for the Feeble-Minded on application to the Secretary, Denison House, 296 Vauxhall Bridge Road, London, S.W., price 1s.

### The Exhibition.

In the corridor of the Council Chamber was arranged an exhibition of work done by the inmates of various homes and institutions for the mentally defective.

The exhibit of the Darenth Industrial Colony, an institution of the Metropolitan Asylums Board, was perhaps the most extensive. The trades taught at Darenth are basketmaking, bootmaking, brushmaking, bookbinding, carpentering, dressmaking, knitting, matmaking, printing, rugmaking, toymaking, tailoring, tinsmiths, upholstering, weaving, and examples of practically all these trades were on view. A full, illustrated account of the work at Darenth appeared in The Hospital of October 11, 1913.

The Stoke Park Colony, Bristol, exhibit, which had been sent up at short notice, was also very interesting. Here were some creditable tapestries, rugs, and various woollen and cotton goods, all materials used being first woven and spun by the children. The Princess Christian's Farm Colony, Hildenborough, Kent, sent flowers, vegetables, butter, eggs, and even chickens as excellent examples of their industry.

Other exhibits, all of them of surprising excellence and utility, came from the Helping Hand Home, 16 Cathcart Hill, N.; the Hastings and St. Leonards Special Residential Schools and Homes; Littleton House School, Newnham Croft, Cambridge; Western Counties Institution for Mental Defectives, Starcross; Bath Home of Help for Women and Girls; Agatha Stacy Homes, Birmingham; North-Eastern Association for the Care of the Feeble-Minded; Cumnor Rise Home for Girls, Oxford, which included, by-the-by, an interesting exhibit of pillow lace; and Scott House, Hitchin, a testing and observation home for "border-line" cases, girls.

